# Extraction and Physicochemical Characterization of a New Polysaccharide Obtained from the Fresh Fruits of *Abelmoschus Esculentus*

**Published:** 2011

**Authors:** Martins Emeje, Christiana Isimi, Stephen Byrn, Joseph Fortunak, Olobayo Kunle, Sabinus Ofoefule

**Affiliations:** a *Department of Pharmaceutical Technology and Raw Materials Development, National Institute for Pharmaceutical Research and Development, Idu, P.M.B.21 Garki-Abuja, Nigeria.*; b *Department of Medicinal Chemistry and Physical Pharmacy, Purdue University, Indiana, USA.*; c * Department of Chemistry, Howard University, USA.*; d *Department of Pharmaceutical Technology and Industrial Pharmacy University of Nigeria, Nsukka, Enugu State, Nigeria.*

**Keywords:** Abelmoschus esculentus gum, Physicochemical characterization, Thermal stability, Sorption profile

## Abstract

This paper is the first multi-scale characterization of the fluidize-dried gum extracted from the fresh fruits of the plant *Abelmoschus esculentus*. It describes the physical, thermal, sorptional and functional properties of this natural gum. Elemental analysis, scanning electron microscopy (SEM), particle size analysis, X-ray powder diffraction (XPRD), thermo-gravimetric analysis (TGA), differential scanning calorimetry (DSC), fourier transmittance infra red (FT-IR), and nuclear magnetic resonance (NMR) spectroscopy were used to characterize the gum sample. *Abelmoschus Esculentus *Gum (AEG) had a glass transition temperature (Tg) of 70°C and no melting peak. It showed a 14.91% loss in weight at 195°C. X-ray diffractogram showed numerous broad halos for AEG. Elemental analysis showed that AEG contains 39.5, 7.3, 51.8, and 1.4% carbon, hydrogen, oxygen and nitrogen respectively. The results obtained in this study established the fundamental characteristics of AEG and suggests its potential application in the food, cosmetic and pharmaceutical sectors.

## Introduction

Excipients are additives used to convert the active pharmaceutical ingredients into dosage forms suitable for administration to patients (1). New and modified excipients continue to emerge with better drug delivery performance; but of particular interest is the increasing trend in research into plant based pharmaceutical excipients (2). Plant products serve as an alternative to synthetic products because of its biocompatibility, non toxicity, biodegradability, environmental-friendly nature and low prices compared to synthetic products. They are generally non-polluting renewable sources for sustainable supply of cheaper pharmaceutical products (1, 2).

Most investigations in drug delivery center on the use of natural gums obtained from plants because of their diverse applications. Natural gums have been employed as disintegrants (3), emulsifying agents (4), suspending agents (5) and binders (6). They have been also found useful in formulating immediate and sustained release preparations (7). 

The plant *Abelmoschus esculentus *is a tall erect annual plant commonly known as ‘Okra’. It is widely cultivated in most tropical countries. It can be grown year round and it is known for its viscous mucilaginous solution in water. This property has been utilized in the production of a plasma expander (8). *Abelmoschus esculentus *gum (AEG) has also been used as suspending and emulsifying agents (4, 5). Recently, the mucilage obtained from Abelmoschus esculentus was reported to have a sustained release property in tablet formulations (7). 

Literature survey revealed limited physicochemical properties of okra mucilage (8). There is no report of a comprehensive physicochemical characterization of the gum. Therefore, the objective of this study was to isolate and undertake a multiscale characterization of the gum in order to establish the fundamental characteristics of AEG and provide insight to its potential use in a variety of fields.

## Experimental

*Materials*

Tragacanth powder (Sigma, UK), fruits of *Abelmoschus esculentus*. All other chemicals and reagents used were of laboratory reagent grade.

*Method*

*Gum extraction*

*AEG *was extracted and purified as previously described by Wang *et a*l. 2006 (3); Endogenous enzymes were inactivated by boiling 100 g of the plant material with 2 L ethanol 80% v/v for 1 h. The plant part was heated in water bath at 90 °C for 2 h to extract the water soluble fraction. The insoluble solids were separated by filtration through a muslin cloth, while the extract (Fraction 1) was centrifuged at 2000 rpm for 10 min to collect the supernatant. The remaining solid after water extraction was extracted with 1 L 0.05 M HCl at 85°C for 30 min, filtered through a muslin cloth. The extract was again centrifuged at 2000 rpm for 10 min and the supernatant provided the acid soluble fraction (Fraction 2). The remaining solids were further extracted with 1 litre 0.05 M NaOH at 85°C for 30 min, filtered through a muslin cloth, centrifuged at 2000 rpm for 10 min and the supernatant collected to yield the alkaline soluble fraction (Fraction 3). The pH of the acid and alkaline soluble fractions was adjusted to 4.5 with NaOH and HCl respectively. All three extracts were precipitated by adding three volumes of 95% ethanol and washed with absolute ethanol three times followed by fluidized-bed drying. The water extract which had the highest yield was selected for further studies.

*Physicochemical characterization of the gum*

*Solubility test*

The separated gum was evaluated for solubility in water, acetone, chloroform and ethanol in accordance with the British pharmacopoeia specifications (9). 

*Swelling index*

The method of Ohwoavworhua and Adelakun 2005 (10) was used. A 1.0 g quantity of each of the samples was placed in each of 15 mL plastic centrifuge tubes and the volume occupied was noted. Ten milliliters of distilled water was added from a 10 mL measuring cylinder and stoppered. The contents were mixed on a vortex mixer (Vortex Gennie Scientific, USA) for 2 min. The mixture was allowed to stand for 10 min and immediately centrifuged at 1000 rpm for 10 min on a bench centrifuge (GallenKamp, England). The supernatant was carefully decanted and the volume of sediment was measured. The swelling index was computed using equation 1:

S = V2 / V1                    (Equation 1)

where S is swelling index, V_1_ is the volume occupied by the gum prior to hydration and V_2_ is the volume occupied by the gum after the hydration.

*Loss on drying*

The method adopted was that specified in the B.P 2004 for acacia (9). A 1.0 g quantity of the sample was transferred into a Petri dish and then dried in an oven at 105°C until a constant weight was obtained. The moisture content was then determined as the ratio of weight of moisture loss to weight of sample expressed as a percentage. 

*Total ash and acid insoluble ash determination*

Ash content was estimated by the measurement of the residue left after combustion in a furnace at 450 °C (10). The ash obtained from the determination of total ash was boiled with 25 mL of 2 M hydrochloric acid solution for 5 min and the insoluble matter filtered and washed with hot water and ignited and weight determined. The acid insoluble percentage ash was calculated (10). 

*pH determination*

This was done by shaking a 1% w/v dispersion of the sample in water for 5 min and the pH determined using a pH meter (Corning, model 10 England) (10).

*Angle of repose*

The static angle of repose, θ, was measured according to the fixed funnel and free standing cone method (10). A funnel was clamped with its tip 2 cm above a graph paper placed on a flat horizontal surface. The powders were carefully poured through the funnel until the top of the cone (h) thus formed just reached the tip of the funnel. The mean diameters (D) of the base of the powder cones were determined and the tangent of the angle of repose was calculated using equation 2:

Tan θ = 2h / D                     (Equation 2)

*Bulk and tap densities*

A 2.0 g quantity of each of the powder samples was placed in a 10 mL measuring cylinder and the volume, V_o_, occupied by each of the samples without tapping was noted. After 100 taps on the table, the occupied volume V_100_ was read. The bulk and tap densities were calculated as the ratio of weight to volume (V_o_ and V_100_ respectively).

*Hausners index*

This was calculated as the ratio of tapped density to bulk density of the samples.

*Compressibility index (C %)*

This was calculated using the following equation:

Compressibility = (Tapped density – Bulk density) / Tapped density × 100                     (Equation 3)

*Thermogravimetric analyses*

Thermogravimetric analyses were performed in a TG apparatus (Shimadzu, Japan). Sample (0.9912 mg) was heated at a rate of 10°C/min from ambient temperature to 200°C. Nitrogen was used as the purge gas at a flow rate of 20 mL/min. 

*Differential scanning calorimetry (DSC) analyses*

Thermal properties of AEG were characterized using a Netzsch DSC 204 F1 Phoenix (Netzsch, Germany). Nitrogen, at the rate of 20 mL/min, was used as purge gas; 2.7 mg of powdered material was sealed in aluminium pan and heated from 30°C up to 400°C at the rate of 10 °C/min, followed by a cooling cycle back to 30 °C at the same rate.

*Fourier transform infrared (FT-IR)*

The FT-IR spectrum of the sample was recorded in an IR spectrometer (Nicolet Magna 4R 560, MN, USA), using potassium bromide (KBr) discs prepared from powdered samples mixed with dry KBr in the ratio of 1 : 200.

*X-ray powder diffraction (XRPD)*

X-ray diffraction patterns of the gum were analyzed using a Siemens D5000 X-ray diffractometer (Siemens, Munich, Germany). Powder sample, packed in rectangular aluminium cells, was illuminated using CuKα radiation (λ = 1.54056 Å) at 45 KV and 40 mA. Samples were scanned between diffraction angles of 5°C to 40°C 2θ. Scan steps of 0.1 were used and the dwell time was 15.0 sec. A nickel filter was used to reduce the K_β_ contribution to the X-ray signal. Triplicate measurements were made at ambient temperature.

*Microstructure studies by SEM*

Morphological features of the gum were studied with a JSM-5600 LV scanning electron microscope of JEOL (Tokyo, Japan). The dried sample was mounted on a metal stub and sputtered with gold in order to make the sample conductive, and the images were taken at an accelerating voltage of 10 KV and at 200 and 2000 x magnification.

*Elemental analysis*

Elemental analysis of carbon, hydrogen and nitrogen was carried using a Leco CHN-2000 determinator. A Perkin-Elmer Elemental Analyzer was used for the determination of oxygen.

*Nuclear magnetic resonance (NMR)*

The NMR spectrum was recorded using a Bruker Spectrometer at 75 MHz for 13C NMR. *AEG *sample was dissolved in an ionic liquid; butylmethylimidazolium chloride and 20% DMSO-d6 by volume to get a signal on which the instrument could lock. The ionic liquid is made as follows: 1-methylimidazole was reacted with *n*-butyl chloride and the alkylation results in an imidazolium ion. The counterion (chloride) is chosen to yield an ionic liquid.

## Results and Discussion

*Physicochemical properties*

The different extraction techniques gave the following yields: 20.01, 0.10 and 6.77% w/w for Water, Acid and alkaline extractions respectively. The water extract was therefore chosen for investigation. 

It was observed that AEG underwent hydration which was over twenty times its original size (Table 1). 

**Table 1:** Some physicochemical characterization of the *AEG *and tragacanth powders.

**Parameters**		**Results**
*Abelmoschus esculentus *gum		Slightly soluble in water. Practically
Solubility		insoluble in ethanol, acetone and chloroform.
Swelling ratio	In 0.1 N HCL	2.3 ± 1.0
	In phosphate buffer 7.4	3.1 ± 1.1
	In water	8.5 ± 1.1
Hydration capacity		18.0 ± 2.0
Moisture sorption ( 100% RH)		67 ± 0.01
Loss on drying ( %)		10.0 ± 0.03
Total ash (%)		0.2 ± 0.04
Acid insoluble ash (%)		0.02 ± 0.01
True density (g/cm^3^)		1.59 ± 0.01
Density of powder	Bulk density (g/cc)	0.219 ± 0.00
	Tapped density (g/cc)	0.326 ± 0.01
Compressibility index (%)		32.82
Hausners quotient		1.49
Angle of repose		45.66 ± 0.61º
pH		6.4 ± 0.05
Flow rate (g/s)		0.17 ± 0.01
Tragacanth gum		Slightly soluble in water. Practically
Solubility		insoluble in ethanol, acetone and chloroform.
Swelling ratio	In 0.1N HCL	6.0 ± 0.11
	In phosphate buffer 7.4	3.4 ± 0.21
	In water	5.8 ± 1.01
Loss on drying (%)		0.67 ± 0.01
Total ash (%)		3.0 ± 0.01
Acid insoluble ash (%)		0.0 ± 0.01
Density of powder	Bulk density (g/cc)	0.76 ± 0.20
	Tapped density (g/cc)	0.77 ± 0.11
Compressibility index (%)		1.30
Hausners quotient		1.01
Angle of repose (º)		20.31 ± 5.01
pH		5.3 ± 0.01

This implies that the gum could be used as binder/disintegrant which on swelling may burst and free up sufficient energy to release drug contents. The extent of swelling for the gum was less than its hydration capacity. The result shows that when used as a binder/disintegrant, *AEG *could absorb sufficient moisture to swell and cause tablets to be disintegrated. Bulk and tapped densities of the gum gave an insight into its flow properties, packaging and arrangement of particles and hence the compactability of the granulation. It was found that the bulk and tapped densities of *AEG *were very low. This may explain the higher swelling capacity of the gum, as water rises by capillary action through pores. This wicking movement of this water through pores of the gum has been found to activate the disintegrant to bring about disintegration and can be exploited by the pharmaceutical industry. Angle of repose (θ) is a function of the internal friction or cohesion of the gum particles. The angle of repose of *AEG *was found to be very high and this shows that *AEG *has more cohesive properties than the reference gum as the value of repose angle has been shown to be high if the powder is cohesive and low if the powder is non-cohesive. It is important to note that different methods may produce different values of the same powder owing to the different way in which the samples were handled prior to measurement. Therefore, angle of repose tend to be variable and not always representative of flow under specific conditions. Adding formulation addictives, such as flow activators (glidants), alteration of processing conditions or using vibration-assisted hoppers or feeders, can improve the flowability of the gum. 

The moisture sorption result shows that the gum sopped 67% moisture at 100% RH. Such knowledge is essential for designing and optimizing many processes involved in production using these gums like drying, packaging, and storing. It is also important in maintaining the quality of formulated drugs using these gums throughout its supposed shelf-life as the moisture sopping up is uneconomical and will with suitable temperature lead to the activation of enzymes that can catalyze the breakdown of medically active drugs. This implies that formulated drugs using this gum should be stored in air tight, moisture impermeable containers.

Table 1 shows some of the physicochemical parameters of both the test and reference gums. The gum extracted from the fresh pods of *Abelmoschus esculentus *is soluble in water and a dispersion of it yielded off a white slimy solution. The gum was practically insoluble in ethanol, acetone and chloroform. Tragacanth which was used as a reference sample, gave a similar solubility profile.

The swelling characteristic of *AEG *was studied in different media; 0.1 N hydrochloric acid, phosphate buffer (pH = 7.4) and water. The swelling was the highest in water followed by phosphate buffer and least in 0.1 N HCl pH. Generally, the results show that the AEG gum has a high swelling index suggesting that the gum may perform well as binder/disintegrant/ matrixing agent. The gum is a pH responsive polymer; it is therefore a smart polymer and may find application in controlled release dosage formulations (11). The relatively higher swelling index obtained for *AEG *at pH = 7.4 implies that unlike tragacanth, the gum may be useful as a matrix former in controlled drug-release. Swelling is a primary mechanism in diffusion controlled release dosage form (12).

The moisture content of *AEG *was low, suggesting its suitability in formulations containing moisture sensitive drugs. The result corroborates the one obtained by TGA. Given suitable temperature moisture will lead to the activation of enzymes and the proliferation of micro organisms, thereby affecting its shelf life. It is important to investigate the moisture content of a material as the economic importance of an excipient for industrial application lies not only on the cheap and easy availability of the biomaterial but the optimization of production processes such as drying, packaging and storing (13).

The total ash and acid insoluble ash value of *AEG *was found to be 2.0 and 1.0% w/w respectively. Ash values reflect the level of adulteration or handling of the drug. More direct adulteration by sand or earth is immediately detected as the total ash which is normally composed of inorganic mixtures of carbonates, phosphates, silicates and silica. Therefore, the low values of total ash and acid insoluble ash obtained in this study indicate that there were low levels of contamination (10).

The bulk and tapped densities give an insight on the packing and arranging the particles and the compaction profile of a material (14). The compressibility index and the angle of AEG repose was 32.82% and 45.66° respectively, implying that the *AEG *has a poor flowability and compressibility, unlike tragacanth with a moderate flowability and compressibility index as indicated by its compressibility value of 1.30% and a repose angle of 20.31º. The knowledge of this is important in scale-up processes involving this material as an excipient in a pharmaceutical formulation. Modification, regarding the improvement in its flow properties during the process development, will be minimal compared to tragacanth. Such process development for optimal production process would include glidants or feeders.

One % w/v suspension of *AEG *gave a pH of 6.4 while that of tragacanth was 5.3. The near neutral pH of *AEG *implies that when used in uncoated tablets, it may be less irritating to the gastrointestinal tract. It may also seem useful to be applied in formulation of acidic, basic and neutral drugs. The pH of an excipient is an important parameter in determining its suitability in formulations since the stability and physiological activity of most preparations depends on pH (15).

*Thermogravimetric analyses*

**Figure 1:**
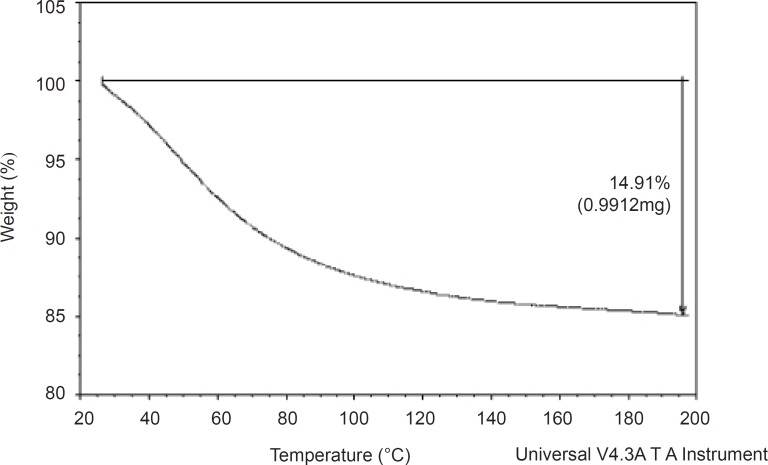
TG curve of *Abelmoschus esculentus *gum

The thermogravimetric (TG) spectrum was used to determine the weight loss of the material on heating. Transitions involving mass changes are detected by TG analysis and this is determined as a function of temperature and time (14). The TG curve for the gum which is shown in Figure 1, demonstrated a one-stage weight loss corresponding to the loss of water around 25-195°C. The curve shows that the gum did not decompose before 200 °C. It has been reported (15) that water is formed by intra- and intermolecular condensation of polymer hydroxyls are the main products of decomposition at temperatures below 300 °C. The gum underwent 14.91% weight loss at 195 °C. This is low, compared to the weight loss reported for other polymers such as starch (15). It implies that *AEG *has a good thermal stability.

*Differential scanning calorimetry (DSC)*

**Figure 2:**
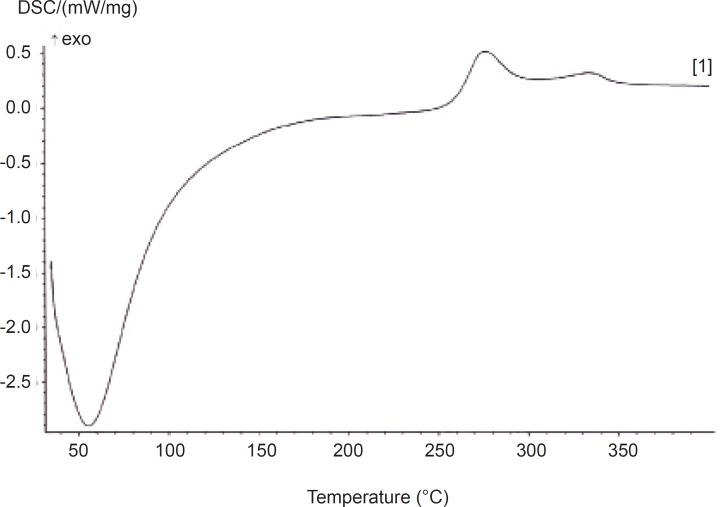
DSC thermogram of *Abelmoschus esculentus *gum

Differential scanning calorimetry (DSC) was used to measure the occurrence of exothermal or endothermal changes with an increase in temperature (Figure 2). 

DSC has been extensively used to study the phase transitions of polymers, owing to its sensitivity and accuracy (16-19). The thermogram for *AEG *is shown in Figure 1 and the corresponding parameters are tabulated in Table 2. 

**Table 2:** Thermal properties of *Abelmoschus esculentus *gum

**Parameter**	***Abelmoschus esculentus *** **gum**
Onset temperature (°C) Peak temperature (°C) Endset temperature (°C) Delta Cp [J/(g*K)] Melting point (°C)	54.9 67.4 79.9 12.5 ------

It shows that the gum exhibits only the amorphous portion. Glass transition (Tg) temperature occurred at 61.5 °C and there was no melting peak. The continuous (broad) endothermic transition that preceded the glass transition is indicative of moisture loss in the sample. The onset peak and conclusion temperatures of phase transition were observed to be very high (Table 2). The glass transition temperature (Tg) was observed to be very low (Table 2). This is contrary to the earlier report (20) which indicates a low degree of crystallinity of the gum. It has also been reported that the materials of low Tg have low crystallinity. High degree of crystallinity has been shown to provide structural stability and made granules more resistant to heat (21-23). This implies that *AEG *may structurally be less stable and less resistant to heat. The knowledge of Tg is essential in production processes and storage since Tg is affected by moisture and other additives, facilitating conversion to the rubbery state and hence facilitating crystallization (15). The gum was also observed to have low enthalpy which is attributed to the presence of a more regular or small and oval granules (24).

*FT-IR*

**Figure 3:**
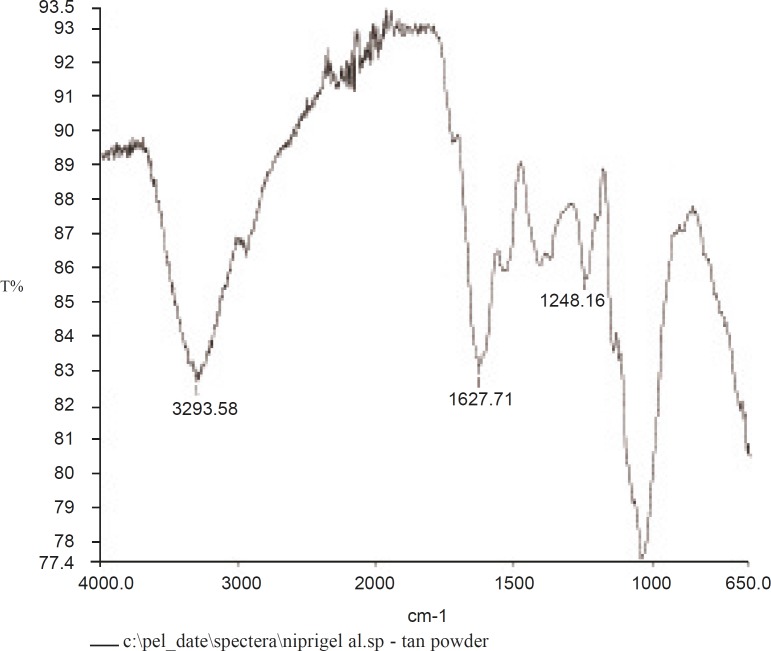
FT-IR spectrum of *Abelmoschus esculentus *gum

The IR spectrum is shown in Figure 3. The finger print region of the spectrum consists of two characteristic peaks between 700 and 1316 cm-^1^, attributed to the C-O bond stretching (25). The band at 1604 cm-^1^ was assigned to the O-H bending of water (26). Contribution from carbonyl stretches in the 1700 cm-^1^ region indicates the presence of ester linkages. Weak stretches in the 1650-1690 cm-^1^ region would mean that this is mostly from lignin.

The absence of significant aromatic stretches in the 1660-1690 cm-^1^ region and the weakness of the stretches, imply that there is a modest amount of cross linking by peptides. The sharp band at 2939 cm-^1^ is characteristic of methyl C-H stretching associated with aromatic rings. The broad band at 3286 cm-^1^ is due to the hydrogen-bonded hydroxyl groups that contribute to the complex vibrational stretches associated with free inter and intra-molecular bound hydroxyl groups which make up the gross structure of carbohydrates (27). This is all consistent with a polysaccharide structure that is neither starch nor cellulose, but has some peptide cross-links and some amino sugars.

*X-ray powder diffraction (XRPD)*

**Figure 4:**
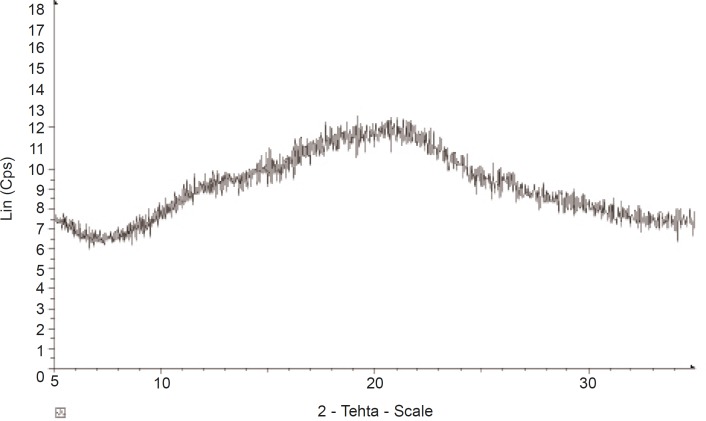
XRD pattern of *Abelmoschus esculentus *gum

The X-ray diffractogram of *AEG *shows presence of numerous halos (Figure 4) with weak peaks, confirming its almost complete amorphous nature. The result of the XPRD confirms that of the DSC which shows that, *AEG *exhibits only an amorphous portion.

*Morphology*

**Figure 5:**
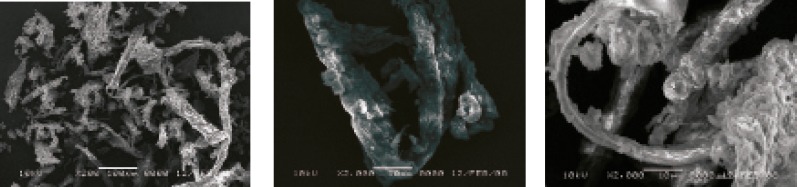
Scanning electron micrograph of *Abelmoschus esculentus *gum

The biological and botanical source of a pharmaceutical material serves as a determining factor in the granule shape, size and morphology. As a result, these characteristics not only help to differentiate between various materials but also give an indication of the processing parameters. The SEM of *AEG *is shown in Figure 5. It exhibits fibrous long non-distinct-shaped large fibers. These properties could be of importance when considering applications based on surface characteristics. The mean particle size was 180 μm. Typical aspect ratio was calculated to be 3 : 2 : 2. The surface area as measured by BET analysis was 1.5 m^2^/g. These properties could be of importance when considering applications based on surface characteristics, for example, use of granules as carrier particles.

*Elemental analyses*

The very high oxygen to carbon ratio of the gums indicate the presence of sugar polymers which are not polymerized into starch since the starch would be in granules which were not present in the SEM. The ratio of carbon to hydrogen is a confirmation that there are no linear carbon chains, but rather carbon in rings sugars indicative of sugars. Elemental analysis of *AEG *indicates the following results : carbon (39.5%), hydrogen (7.3%), oxygen (51.8%), nitrogen (Kjeldahl analysis) 1.4%. Atomic absorption confirmed C, H, N, and O and also indicated traces of sulfur and bromine. 

^13^ *C NMR of AEG*

**Figure 6:**
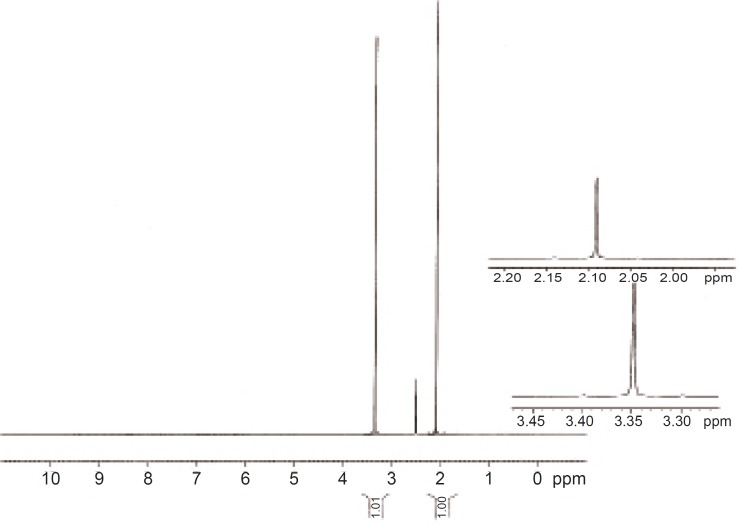
NMR spectrum of *Abelmoschus esculentus *gum

A good 1H NMR spectra was not obtained for *AEG*. It was insoluble even in DMSO-d6 and a meaningful spectrum was not possible. 13C NMR was however successful (Figure 6). While 1H NMR shows up the protons, 13C NMR shows signals for individual carbons. The large peaks in the 13C spectrum that are shown full scale in the scan demonstrate the ionic liquid carbons. The blow up region between about 55 ppm and 110 ppm indicates the peaks that can be seen in the spectrum which are totally resolved from the ionic liquid. The peaks in this range all show up in the region where carbons bonded to heteroatoms – most likely oxygen should be seen. All of these indicate that the polymers are highly oxygenated. 

## Conclusions

The results obtained in this study establish the fundamental characteristics of okra gum and recommend further exploration of its potential for use in a variety of fields. The large particle size, along with high swellability indicates its usefulness in food and cosmetic industry. The weak associative forces stabilizing the okra gum as shown by the DSC result could be explored for its potential use as a disintegrant in the pharmaceutical sector. Further work will continue to develop insights to the various applications of okra gum.
